# Nanostructured fibers as a versatile photonic platform: radiative cooling and waveguiding through transverse Anderson localization

**DOI:** 10.1038/s41377-018-0033-x

**Published:** 2018-07-18

**Authors:** Norman Nan Shi, Cheng-Chia Tsai, Michael J. Carter, Jyotirmoy Mandal, Adam C. Overvig, Matthew Y. Sfeir, Ming Lu, Catherine L. Craig, Gary D. Bernard, Yuan Yang, Nanfang Yu

**Affiliations:** 1 Department of Applied Physics and Applied Mathematics, Columbia University, New York, NY 10027 USA; 2 Center for Functional Nanomaterials, Brookhaven National Laboratory, Upton, NY 11973 USA; 3 Museum of Comparative Zoology, Harvard University, Cambridge, MA 02138 USA; 4 Department of Electrical Engineering, University of Washington, Seattle, WA 98195 USA

## Abstract

Broadband high reflectance in nature is often the result of randomly, three-dimensionally structured materials. This study explores unique optical properties associated with one-dimensional nanostructures discovered in silk cocoon fibers of the comet moth, *Argema mittrei*. The fibers are populated with a high density of air voids randomly distributed across the fiber cross-section but are invariant along the fiber. These filamentary air voids strongly scatter light in the solar spectrum. A single silk fiber measuring ~50 μm thick can reflect 66% of incoming solar radiation, and this, together with the fibers’ high emissivity of 0.88 in the mid-infrared range, allows the cocoon to act as an efficient radiative-cooling device. Drawing inspiration from these natural radiative-cooling fibers, biomimetic nanostructured fibers based on both regenerated silk fibroin and polyvinylidene difluoride are fabricated through wet spinning. Optical characterization shows that these fibers exhibit exceptional optical properties for radiative-cooling applications: nanostructured regenerated silk fibers provide a solar reflectivity of 0.73 and a thermal emissivity of 0.90, and nanostructured polyvinylidene difluoride fibers provide a solar reflectivity of 0.93 and a thermal emissivity of 0.91. The filamentary air voids lead to highly directional scattering, giving the fibers a highly reflective sheen, but more interestingly, they enable guided optical modes to propagate along the fibers through transverse Anderson localization. This discovery opens up the possibility of using wild silkmoth fibers as a biocompatible and bioresorbable material for optical signal and image transport.

## Optical fibres: Unique properties woven in silk

Silk cocoon fibres of the comet moth *Argema mittrei* could be used to create new materials for transporting optical signals and images, possibly serving as biocompatible and resorbable light guides for medical applications. Researchers led by Nanfang Yu at Columbia University in New York, explored the unique optical properties of the silk. The fibres have many filamentary air voids arranged in a dense array that effectively scatters sunlight. This allows them to act as natural cooling devices, protecting the moth pupae from temperature fluctuations. The researchers were inspired to make synthetic fibres mimicking the natural ones. In addition to the useful radiative cooling effect, the fibres guide light in a manner that may allow efficient transmission of signals and images. Applications in optical therapies, medical diagnostics and tissue engineering should be explored.

## Introduction

Silkworm cocoon fibers are remarkable natural materials that protect pupae from rapid temperature fluctuations, ultraviolet (UV) radiation, and predatory attacks^1–5^. These exceptional thermal, optical, and mechanical properties, combined with biocompatible and biodegradable properties, make silk fibers an ideal candidate for tissue engineering and other biomedical applications^6–10^. This study aims to build and expand upon the silk fiber’s functionalities by exploring new applications in radiative cooling, light delivery, and image transport.

**Fig. 1:**
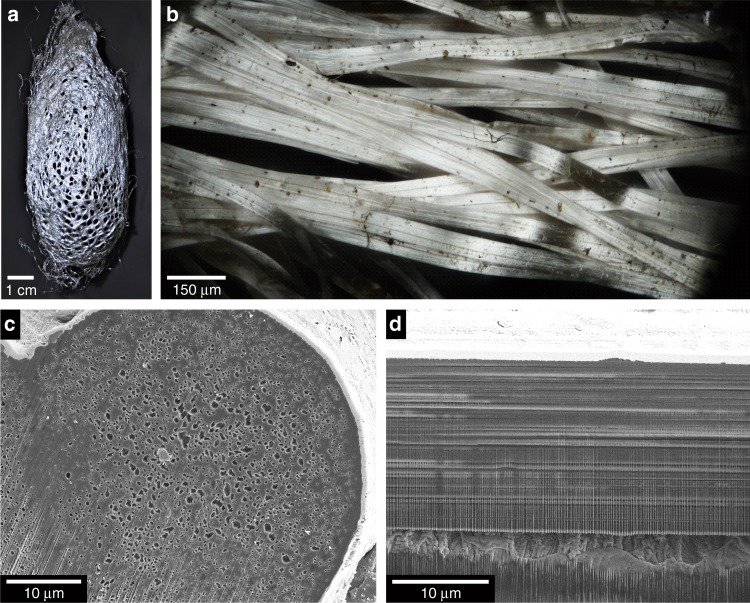
Morphology of the cocoon and silk fibers of the comet moth. **a** Photograph of a comet moth cocoon, showing its reflective sheen. **b** Dark-field optical microscopy image showing overlapping cocoon fibers. **c** Scanning electron microscopy (SEM) image of the transverse cross-section of a comet moth silk fiber prepared by focused ion beam (FIB) milling. **d** SEM image of the longitudinal cross-section of a silk fiber prepared by FIB milling.

The moth species whose cocoon fibers were examined in this study is *Argema mittrei*, commonly known as the comet moth. The moth is one of the largest in the world, with cocoons spanning 6–10 cm in length^11^. Under sunlight, the cocoons, as well as individual silk fibers that make up the cocoons, exhibit a bright, silvery, metallic sheen (Fig. 1a, b). While diffuse reflection in randomly structured materials is often observed in nature^12–15^, light reflection with the high degree of specularity observed in these moth cocoon fibers is unique for a natural biological system.

As demonstrated in this study, the unique optical properties of these fibers are the result of filamentary air voids propagating along the cocoon fibers. The voids have cross-sectional sizes comparable to wavelengths of visible and near-infrared light and thus act as scattering centers that enhance the solar reflectance of the fibers. Furthermore, the variety of chemical bonds of the silk proteins leads to high emissivity in the mid-infrared. The combined effect of high solar reflectance and thermal emissivity enables the cocoons to regulate temperature via passive radiative cooling^16–19^. Drawing inspiration from the structure and optical properties of these natural fibers, we fabricated biomimetic fibers embedded with a high density of voids and characterized their radiative-cooling capabilities through spectroscopic measurements. In addition to enabling radiative cooling, cocoon fibers with longitudinally invariant voids possess the ability to guide light along their longitudinal direction through transverse Anderson localization. The latter is a phenomenon first observed experimentally in 2007 in a photorefractive crystal, in which small, random one-dimensional (1D) perturbations of optical refractive indices were introduced through an optical nonlinear effect^20,21^. Since then, researchers have been able to create fibers containing longitudinally invariant random structures using various fiber drawing techniques with different materials systems and incorporate them into applications in image transport, light focusing, secure information transport, and random lasers^22–27^. This study discusses the first experimental observation of transverse Anderson localization in a natural biological system, and demonstrates potential applications in light guiding, image transport, and light focusing.

## Results

### Morphology and optical properties of comet moth cocoon fibers

The comet moth cocoon is made of threads, each consisting of a pair of fibers bonded by a coating of sericin^28^. We used focused ion beam (FIB) milling to expose the fiber’s transverse and longitudinal cross-sections. The transverse cross-section of one fiber (Fig. 1c) shows that it has a diameter of ~40 µm and is populated with irregularly shaped voids with sizes ranging from hundreds of nanometers to one micron (Supplementary Information Section 2). Small voids tend to be located toward the edge of the fiber and large voids toward the center. The region where sericin joins the two fibers is free of voids. The longitudinal cross-section of a fiber (Fig. 1d) shows that the voids propagate for at least tens of microns without varying in size.

**Fig. 2:**
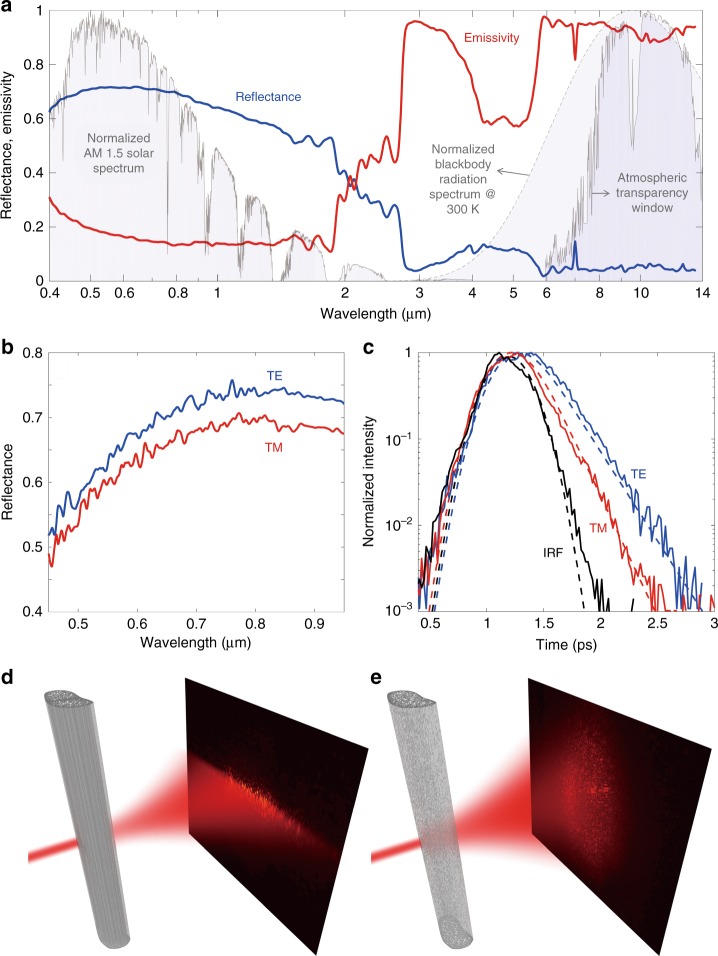
Optical characterization of single cocoon fibers. **a** Integrated hemispherical reflectance and emissivity (calculated by 1−reflectance−transmittance) spectra of a single comet moth cocoon fiber from the visible to the mid-infrared (*λ* = 400 nm–13.5 μm). The normalized spectral intensity of the AM 1.5 solar spectrum, the blackbody radiation spectrum at 300 K, and the atmospheric transparency window are plotted in the background. **b** Integrated hemispherical reflectance spectra of a single cocoon fiber illuminated with transverse electric (TE) and transverse magnetic (TM) polarized light at normal incidence, where TE polarization is defined with the electric field aligned with the longitudinal direction of the fiber. **c** Time-of-flight measurements of a single cocoon fiber. IRF represents the instrument response function, which is the cross-correlation of ultra-short reference (*λ* = 800 nm) and probe (*λ* = 600 nm) pulses. Cross-correlation between the reference pulse and a TE polarized probe pulse passing through the fiber (blue curve) shows a longer decaying tail compared with that in the case of TM polarization (red curve), indicating that TE polarized light interacts more strongly with the nanostructured fiber. Dashed curves are fits to the experimental data (solid curves) to extract photon lifetimes. **d** Schematic showing a focused laser beam at *λ* = 633 nm passing through a single cocoon fiber oriented in the vertical direction. Measured scattering pattern is shown on the right. Filamentary voids along the fiber prevents excessive scattering in the vertical direction; thus, the scattering pattern forms a horizontal narrow band. **e** Schematic showing the focused laser beam passing through a regenerated silk fiber bundle (as a control) containing a high density of nanoscale particulate voids (Fig. 3b). Measured scattering pattern on the right shows that there is no preferential scattering direction due to the 3D nature of the voids

Directional-hemispherical reflectance measurements performed on single silk fibers measuring ~50 µm thick show that single fibers have a high reflectance of 0.66 normalized to the solar spectrum (Fig. 2a). The fibers’ strong reflectance enhancement is the result of multiple light-scattering events caused by the random voids inside the fibers, where the void sizes are comparable to the wavelengths of sunlight. At longer wavelengths, however, as the voids become subwavelength in size, they no longer act as strong scattering centers, and reflectance is greatly reduced. In fact, the fibers become highly absorptive in the mid-infrared range (*λ* = 6–14 µm) due to strong and broadband absorption of a variety of chemical bonds of fibroin proteins that comprise the silk fibers. The wavelength range over which infrared absorptivity is enhanced overlaps well with the atmospheric transparency window (*λ* = 8–14 µm) and the blackbody radiation spectrum of warm objects^29,30^. Enhanced absorptivity in the mid-infrared enable the cocoon fibers to reach a high emissivity of 0.88, weighted by the thermal radiation spectrum at 300 K. Thus, the portion of solar energy absorbed by the cocoon can be efficiently dissipated back to the environment through thermal radiation. The combined effects of high solar reflectance and high thermal emissivity help prevent the pupa inside a cocoon from overheating when the cocoon is under direct sunlight.

To further understand how these filamentary voids affect the optical properties of the fibers and to understand the specular reflection of these fibers in the visible, we shone linearly polarized light onto single silk fibers and measured spatial, spectral, temporal, and polarization-dependent properties of scattered light. The integrated reflectance of the silk fiber was higher when illuminated with transverse electric (TE) polarized light than when illuminated with transverse magnetic (TM) polarized light (Fig. 2b). Here, TE corresponds to the electric field parallel to the longitudinal direction of the fiber. This difference in reflectivity is the result of form birefringence created by the filamentary voids, as confirmed through finite-difference time-domain (FDTD) simulations (Supplementary Information Section 4). These filamentary voids also enable the silk fibers to exhibit a strong specular sheen: the 1D nature of these voids limits the scattered light to within a narrow angular range in the far field. The effect was demonstrated by imaging the forward scattering pattern of a laser beam at *λ* = 633 nm focused onto a single fiber. Figure 2d shows that the far-field scattering pattern of a single cocoon fiber is a narrow horizontal stripe perpendicular to the vertically oriented fiber, indicating highly directional scattering as a result of the filamentary voids. By contrast, a regenerated silk fiber of similar width and thickness but filled with three-dimensional (3D) voids produces a diffuse scattering pattern with no preferential scattering direction (Fig. 2e).

The scattering strength of these fibers can be quantified by a cross-correlation measurement technique (Supplementary Information Section 5)^12^. We measured the temporal profile of an ultrashort laser pulse at *λ* = 600 nm before and after it passed through a single cocoon fiber along the transverse direction (Fig. 2c). The temporal profile before passing through the cocoon fiber was used as the instrument response function (IRF). The IRF was convolved with an exponential decay function to fit and extract the photon lifetime, which positively correlates with the strength of light scattering inside the random structures of the cocoon fiber. The measured photon lifetime was 210 fs for TE polarized light and 155 fs for TM polarized light. The results agree qualitatively with the polarization-dependent reflectance measurements (Fig. 2b) and FDTD simulation results (Supplementary Information Section 4), which show that TE-polarized light interacts more strongly with the filamentary voids than does TM polarized light.

### Regenerated silk and PVDF biomimetic fibers for radiative cooling

We demonstrated that fibers of the comet moth possess passive radiative-cooling capabilities. The capabilities, however, are limited by materials absorption in the solar spectrum and the density of voids (Fig. 2a; Supplementary Information Section 2). Drawing inspiration from the natural silk fibers, we explored alternative materials of choice and fiber-pulling techniques to create biomimetic fibers with optimized radiative-cooling capabilities.

**Fig. 3:**
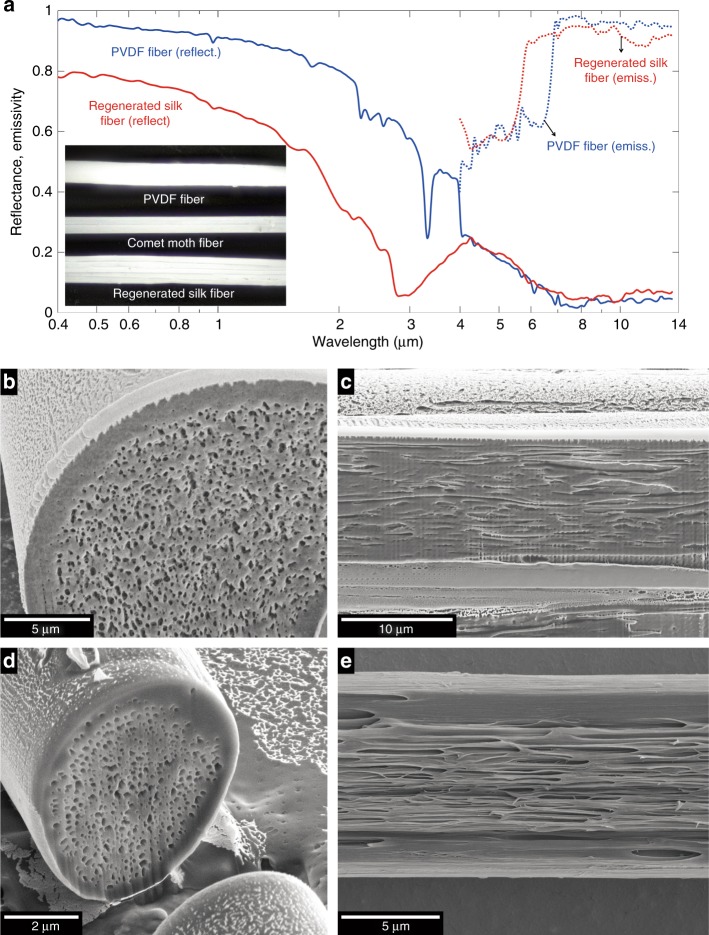
Biomimetic fibers with a high density of internal voids for radiative cooling. **a** Integrated hemispherical reflectance and emissivity spectra of a ~100-μm-thick bundle of regenerated silk fibers and a single PVDF fiber measuring ~100 μm in diameter from the visible to the mid-infrared region. Inset shows a photograph of a nanostructured PVDF fiber, a bundle of nanostructured regenerated silk fibers, and a silk thread of the comet moth. **b**, **c** SEM images of transverse and longitudinal cross-sections, respectively, of a regenerated silk fiber containing a high density of voids. **d**, **e** SEM images of transverse and longitudinal cross-sections, respectively, of a PVDF fiber containing a high density of voids

We obtained regenerated silk fibroin from cocoons made by the domestic silk moth, *Bombyx mori* (Supplementary Information Section 7)^31^. By chemically removing sericin, regenerated fibroin offers substantially reduced absorption in the visible and near-infrared part of the solar spectrum^32^, while providing absorption properties similar to those of natural silk fibers in the mid-infrared region. Researchers have demonstrated that fibers extruded through wet spinning from a solution of regenerated fibroin can have a greater tensile strength than do natural *Bombyx mori* fibers^33^. We modified the fiber spinning recipes reported in the literature^34^, in particular, the concentrations of the silk fibroin solution and the coagulation bath, to introduce voids inside the wet-spun fibers and to control their density (Fig. 3b; Supplementary Information Section 7). We found that a silk fibroin concentration of 13.9% yields the highest density of voids. The regenerated silk fibroin fibers were first spun onto a motorized drum at a controlled speed to achieve a thickness of a few tens of microns. The voids were then stretched into a filamentary form (Fig. 3c) through a drawing process by a second motorized drum rotating two to four times faster than the first one. Spectral measurements of ~100-μm-thick bundles of regenerated silk fibers with a high density of voids showed that the fiber bundle had an integrated solar reflectance of 0.73 and an integrated thermal emissivity of 0.90 (Fig. 3a).

While the fibers made from regenerated fibroin provide good optical properties, silk fibroin’s proneness to long-term UV radiation, water and heat damage and its cost as a raw material limit its applications in radiative-cooling applications^35,36^. A widely available thermoplastic, polyvinylidene difluoride (PVDF), which is highly resistant to UV radiation, heat and water damage and exhibits low absorption in the solar spectrum, was explored as an alternative material^37,38^. By properly choosing the solvent, the ratio between PVDF and the solvent, and the coagulation bath, we were able to pull PVDF fibers containing 3D voids (Fig. 3d; Supplementary Information Section 7; our related work on creating hierarchically porous PVDF coatings that exhibit high-performance passive daytime radiative cooling will be reported elsewhere.). A similar drawing process was used to stretch the voids into a filamentary form (Fig. 3e). Optical measurements performed on pre-drawn thicker fibers, ~100 μm in diameter, showed that the fibers had a high solar reflectance of 0.93 and a high thermal emissivity of 0.91 (Fig. 3a). We note that our regenerated silk and PVDF biomimetic fibers both exhibited higher solar reflectance compared with the comet moth cocoon fiber; this enhancement is primarily due to the higher density of voids created in the biomimetic fibers (i.e., 5.5 voids/μm^2^ for the regenerated silk fiber, 17 voids/μm^2^ for the PVDF fiber, and 2.2 voids/μm^2^ for the comet moth cocoon fiber; Supplementary Information Section 2). Studies comparing the solar reflectance spectra of regenerated silk and PVDF fibers with low and high void concentrations were also conducted (Supplementary Information Section 9), which further confirmed that the high solar reflectance observed in these fibers was the result of a high density of voids. We note that the filamentary voids in our biomimetic fibers were not as long as those observed in the comet moth cocoon fibers. The natural silk fibers’ amazing ability to maintain longitudinal invariance motivated us to investigate light propagation along these nanostructured fibers confined by strong light scattering in the transverse direction.

### Transverse Anderson localization in comet moth fibers

Anderson localization in 3D systems requires a critical level of scattering strength, quantified by the Ioffe–Regel criterion, which can be satisfied in high-refractive-index contrast materials systems^39^. However, the scaling theory of localization dictates that Anderson localization will always occur in random two-dimensional coupled waveguide arrays, even for low-refractive-index contrast systems^40^. In the case of transverse Anderson localization, a beam first undergoes diffusive broadening as it propagates along the longitudinal direction of the waveguide array but ultimately reaches a mean localization radius, called the localization length *ξ*, as it propagates further down the array. The onset of transverse localization can be characterized by the exponentially decaying tail of the beam’s transverse intensity profile. The localization length *ξ* of a guided light beam and the mean free path l^*^ of photons propagating in the nanostructured fibers can be estimated by using the following equations:

1 $$I \sim {\mathrm{exp}}\left( { - 2\left| r \right|/{\mathrm{\xi }}} \right)$$

2 $${\mathrm{\xi = }}l^ \ast {\mathrm{exp}}\left( {{\pi k}_ \bot l^{ \ast 2}/2} \right)$$

where *I* is the beam intensity profile, *r* is the distance from the beam center, *k*_⊥_ = 2/*ω*_o_ is the transverse wavenumber, and *ω*_o_ is the initial width of the beam at the entrance facet of the fiber^20^.

**Fig. 4:**
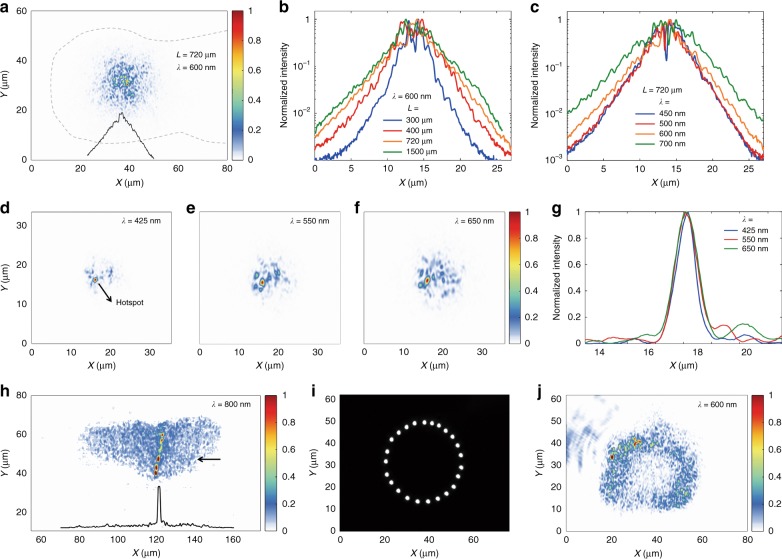
Transverse Anderson localization in comet moth cocoon fibers. **a** Intensity distribution of a beam at *λ* = 600 nm under transverse localization exiting the end facet of a cocoon fiber measuring ~720 μm in length. The dashed curve shows the outer edge of the fiber. The black curve in the figure shows the logarithm of the averaged intensity profile of the beam. **b** Logarithm of averaged intensity profiles at the exit facet for fibers of different lengths with *λ* = 600 nm. **c** Logarithm of averaged intensity profiles at the exit facet for a fiber with length *L* = ~720 μm at different wavelengths. **d**–**f** Intensity distributions showing a highly localized hotspot in a fiber with length *L* = ~150 μm at three wavelengths. **g** Profiles of the hotspot at the three wavelengths. **h** Intensity distribution showing light being guided by a sericin slab region between two cocoon fibers. The black curve shows the profile of the guided mode at a location indicated by an arrow. **i** Optical image of a ring of 1-μm apertures used for image transport through a cocoon fiber with length *L* = ~400 μm. **j** Intensity distribution at the exit facet of the fiber showing the transport of the ring pattern

We characterized how a light beam broadens and ultimately reaches full confinement as a result of transverse Anderson localization in comet moth cocoon fibers. A set of fiber segments with different lengths (*L* = 300, 400, 720, and 1500 μm) were cut and the facets polished using FIB milling. All fiber segments were from the same fiber and cut next to one another to minimize variations in their cross-sectional void pattern. A high-numerical-aperture (NA = 0.55) objective was used to launch a focused beam toward one facet of a fiber segment, and the exit facet was imaged with a matching objective to characterize the beam upon exiting the segment. Figure 4a shows the intensity distribution at the exit facet of a fiber segment (*L* = 720 μm, *λ* = 600 nm). The black curve in Fig. 4a shows the logarithm of the average intensity profile through the center of the beam, and its linear slopes are an indication of transverse Anderson localization. The intensity profiles for the four fiber segments of different lengths (Fig. 4b) show the evolution of the beam profile from initial diffusive broadening to eventual full confinement as *L* increases. Figure 4c shows the intensity profiles for the fiber segment with *L* = 720 μm at various wavelengths (*λ* = 450, 500, 600, and 700 nm). The figure shows that, while the beam remains localized with increasing wavelength (at least up to *λ* = 850 nm), the localization length *ξ* increases with wavelength. The localization length *ξ* of the fiber at *λ* = 600 nm, for example, can be estimated by fitting the exponentially decaying tail of the intensity profile with Eq. (1), yielding *ξ* = 4.6 μm, which is smaller than that reported in a recent study utilizing a man-made high-refractive-index contrast system of glass and air^41^. In a recent work, Choi et al.^42^ explored localized modes in domestic silkworm fibers by utilizing transmission matrix measurements and by exciting the modes in the fibers embedded with gain media. Their theoretically calculated localization length, *ξ* = 4.5 μm, and experimentally measured average mode size of 4.2 μm are similar to our experimentally measured values. The mean free path l^*^ at *λ* = 600 nm can be calculated using Eq. (2), where the entrance beam size *ω*_o_ is ~2 μm, yielding l^*^ ~ 0.98 μm, which is significantly smaller than the sizes reported in early demonstrations of transverse Anderson localization with low-refractive-index contrast systems^20,24^.

The small localization length compared with the transverse size of the fibers enables the fiber system to transport simple patterns. Figure 4i shows an optical image of a series of apertures measuring 1 μm in diameter, milled using FIB in a gold thin film and forming a 30-μm-diameter ring. The aperture pattern was butt coupled to the entrance facet of a fiber segment of length 400 μm and illuminated with a large-diameter beam at *λ* = 600 nm. The image at the exit facet of the fiber segment (Fig. 4j) is a discernible ring pattern, with a resolution limited by the localization length of the system.

As the position of the fiber facet moves with respect to the input beam, light can sometimes be tightly confined in certain regions of the fiber that are free of voids and surrounded by a high density of scattering centers. Figure 4d–g shows one of these confined hotspots, where the confinement is maintained as the wavelength varies from 425 to 650 nm. The full-width at half-maximum (FWHM) sizes of the hotspot at *λ* = 425, 550, and 650 nm are 0.75, 0.94, and 1 μm, respectively, which are smaller than the spot size of the entrance beam (~2 μm). The focusing abilities of the cocoon fibers have the potential to be further enhanced through wavefront shaping and optimization with adaptive optics^22^. In addition to waveguiding through transverse Anderson localization, Fig. 4h shows that these silk fibers can also act as a slab waveguide: the central sericin region free of voids can act as a waveguide core, and lateral confinement is provided by scattering centers in the fibers.

It is crucial to note that the silk materials exhibit intrinsic absorption in the visible spectrum (sericin being more absorptive than fibroin at shorter wavelengths) and that the filamentary voids in fact slowly morph along the longitudinal direction. Consequently, the interesting optical properties and potential applications discussed above related to transverse Anderson localization cannot be realized in fibers longer than a few millimeters. Reduction in propagation losses, however, is possible by chemically removing the sericin coating surrounding silk fibers^43^.

## Discussion

We studied the optical properties of nanostructured silk fibers of the comet moth. We characterized the one-dimensional nature of these voids and their scattering strength through polarization-dependent reflectance measurements, time-of-flight measurements, and far-field scattering measurements. We found that the silk fibers exhibit radiative-cooling capabilities; spectroscopic studies show that strong back-scattering of the nanostructured voids in the visible and near-infrared regions enhances solar reflectance and intrinsic materials absorption of fibroin in the mid-infrared region enhances thermal emissivity. Drawing inspiration from the natural system, we spun biomimetic fibers using regenerated silk fibroin and PVDF, and showed that they possess exceptional optical properties for radiative-cooling applications. Furthermore, for the first time, we observed transverse Anderson localization in a natural biological fiber system: highly confined waveguide modes with localization length as small as *ξ* = 4.6 μm can propagate along silk fibers of the comet moth, and waveguiding is enabled by strong light scattering in the transverse direction. These silk fibers have the potential to be used for delivering light and transporting images in situations in which the fiber must be biocompatible and bioresorbable. Future work may benefit from investigating the mechanism behind the fiber-pulling process utilized by wild silk moths that create silks with filamentary voids and emulating the process to create bioinspired fibers with longitudinally invariant voids.

## Materials and methods

For the visible and near-infrared portion of the spectrum, reflection and transmission measurements were carried out using a Fourier transform-based spectrometer (Bruker Vertex 80v) equipped with a laser-stabilized high-brightness xenon plasma light source (Energetiq eq-99). Forward and backward scattered light was captured with a 2-inch visible/near-infrared integrating sphere (Thorlabs IS200-4), coupled with a set of silicon and indium gallium arsenide detectors. The integrating sphere wall material was used to calibrate the measurements. For the measurements in the mid-infrared portion of the spectrum, a Fourier transform infrared spectrometer (Bruker Vertex 70v), a 2-inch integrating sphere (Labsphere Model 4P-GPS-020-SL) coated with diffuse gold reflectors and a mercury cadmium telluride detector were used. Further details regarding the following subjects can be found in the Supplementary Information: focused ion beam milling of nanostructured fibers; void size, density and distribution; reflection/transmission measurement; finite-difference time-domain simulations; time-of-flight measurements; far-field scattering pattern characterization of single cocoon fibers; fabrication of biomimetic fibers using silk fibroin and PVDF; characterization of fibers supporting transverse Anderson localization; and solar reflectance study of biomimetic fibers with high and low void concentrations.

## Electronic supplementary material

Supplementary material
