# Supplementary material for: Nanostructured fibers as a versatile photonic platform: radiative cooling and waveguiding through transverse Anderson localization
Source: Light Sci Appl. 2018 Jul 18;7:37. doi: 10.1038/s41377-018-0033-x (PMC6107007; doi:10.1038/s41377-018-0033-x)
Supplement: Supplementary file 1 — Supplementary material [file 41377_2018_33_MOESM1_ESM.docx]

Supplementary Information

**Nanostructured fibres as a versatile photonic platform: Radiative cooling and waveguiding through transverse Anderson localization**

Norman Nan Shi^1^, Cheng-Chia Tsai^1^, Michael J. Carter^1^, Jyotirmoy Mandal^1^, Adam C. Overvig^1^, Matthew Y. Sfeir^2^, Ming Lu^2^, Catherine L. Craig^3^, Gary D. Bernard^4^, Yuan Yang^1^, and Nanfang Yu^1^*

^1^Department of Applied Physics and Applied Mathematics, Columbia University, New York, NY 10027, U.S.A.

^2^Center for Functional Nanomaterials, Brookhaven National Laboratory, Upton, NY 11973, U.S.A.

^3^Museum of Comparative Zoology, Harvard University, Cambridge, MA 02138, U.S.A.

^4^Department of Electrical Engineering, University of Washington, Seattle, WA 98195, U.S.A.

**1. Focused ion beam milling of nanostructured fibers**

The cross-sectional images of the natural and manmade fibers were obtained with a focused ion beam/scanning electron microscopy dual system (FEI Helios NanoLab DualBeam). A layer of 500-nm Platinum was first deposited on top of a fiber at the position of the cross-sectional cut to protect the fiber from milling-induced damages. A high current ion beam (Ga+, 30 KV, 21 nA) was then used to cut through the fiber and expose its cross-section, followed by using a smaller current ion beam (30 kV, 2.8 nA) to polish the cross-sectional surfaces. Similar ion beam milling conditions were used to prepare the end facets of cocoon fibers for transverse Anderson localization experiments. Platinum protective coating was not used in this case.

**2. Void size, density and distribution**

Cross-sectional scanning electron microscopy (SEM) images of the comet moth fibers were binarized by utilizing the image contrast between the voids and the surrounding solid fibroin regions (Fig. S1b). An image processing software, ImageJ, was then used to obtain the sizes of all the voids inside the fiber. The histogram of void sizes is shown in Fig. S1c. The diameters of the voids are shown to range from a few tens of nanometers to about a micron. The average void size is 236 nm, the void density is 2.2 voids/μm^2^, and the air-void filling fraction is 9.8% for this particular fiber. Similar processing techniques were used in the case of biomimetic regenerated silk and PVDF fibers, where the average void diameter is 145 nm and 106 nm, the void density is 5.5 and 17 voids/μm^2^, and the air-void filling fraction is 14.5% and 17.7%, respectively.


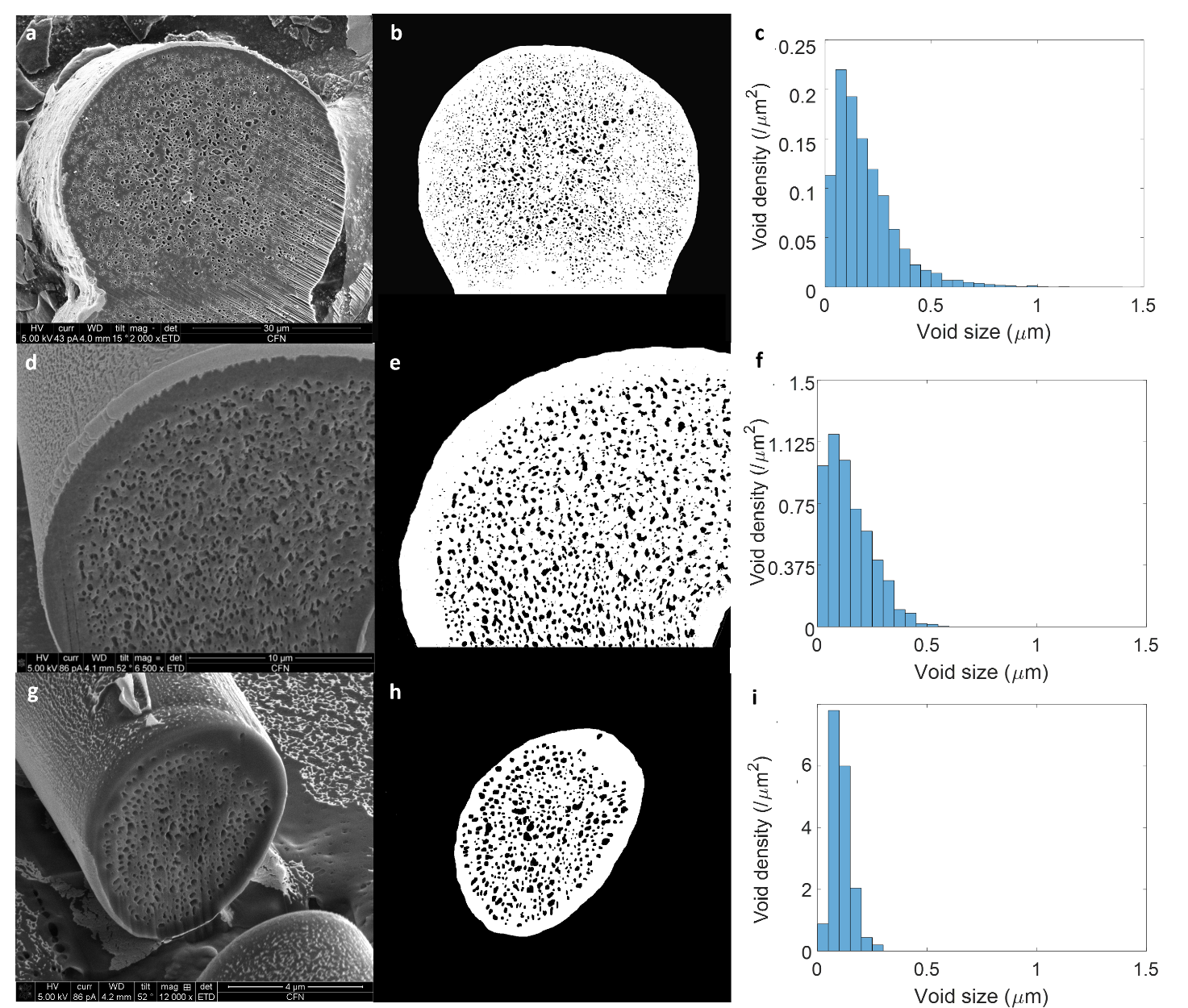


**Figure S1.** First column: SEM images of the transverse cross-section of a comet moth fiber (top), a regenerated silk fiber (middle), and a biomimetic PVDF fiber (bottom). Second column: Binarized images of the SEM images outlining all the voids. Third column: Histograms showing the size distributions of the voids. Summation of all the bars in a histogram yields the total void density.

**3. Reflection/transmission measurement**

In order to account for reflectance of the fibers with respect to solar radiation, we defined:

$\bar{R}_{solar}= \frac{\int_{\lambda_{1}}^{\lambda_{2}} R(\lambda)M(\lambda)d\lambda}{\int_{\lambda_{1}}^{\lambda_{2}} M(\lambda)d\lambda}$ (S1)

where $\bar{R}_{solar}$ is the hemispherical reflectance normalized to the AM 1.5 Global solar intensity spectrum as a function of wavelength, $M(\lambda)$.^[1]^ λ_1_ =400 nm and λ_2_ =2.5 μm are the lower and upper limits of spectra measured by our visible/near-infrared Fourier-transform spectrometer. $R(\lambda)$ is the measured hemispherical reflectance as a function of wavelength. The obtained value $\bar{R}_{solar}$ characterizes the percentage of sunlight reflected by the fibers.

Similarly, we defined integrated hemispherical emissivity as:

$\bar{\varepsilon}_{T=300 K}= \frac{\int_{\lambda_{1}}^{\lambda_{2}} \varepsilon\left( \lambda\right){I\left( T, \lambda\right)}_{blackbody}d\lambda}{\int_{\lambda_{1}}^{\lambda_{2}} I\left( T, \lambda\right)_{blackbody}d\lambda}$ (S2)

where $\varepsilon(\lambda)$ is the measured hemispherical emissivity as a function of wavelength, λ_1_ =6 μm and λ_2_ =14 μm are the lower and upper limits of spectra measured by our Fourier-transform infrared spectrometer, and ${I\left( T, \lambda\right)}_{blackbody}$ is the spectral intensity of a blackbody at *T* = 300 K. The obtained value $\bar{\varepsilon}_{T=300 K}$ characterizes the ability of the fibers to dissipate heat through thermal radiation.

**4. Finite-difference time-domain simulations**

Finite-difference time-domain (FDTD) (Lumerical FDTD solutions) simulations were conducted to investigate reflection of the cocoon fiber as a function of incident polarization. A transverse cross-sectional SEM image of a comet moth fiber was imported into the software to obtain the structure used in FDTD simulations (Fig. S2a). The voids were assumed to be invariant in the longitudinal direction of the fiber. A refractive index of n = 1.5 was assigned to the material at all wavelengths,^[2]^ and no absorption was considered in the simulations.

The simulated reflectance spectra show that reflection of TE polarized incident light is higher than that of TM polarized light (Fig. S2b). It is also observed that as the wavelength of light increases towards the near-infrared, reflectance decreases. This trend matches the measurement results, which show that the scattering strength of the fiber material decreases as the wavelength of light becomes substantially larger than the size of the voids.

The simulated temporal profile of an ultra-short pulse passing through the cocoon fiber along the transverse direction (Fig. S2c) confirms the time-of-flight measurement results, which show that TE polarized light interacts stronger with the voids, leading to a more elongated tail of the pulse. Photon lifetimes estimated from the simulations are 270 fs for TE polarized light and 230 fs for TM polarized light.


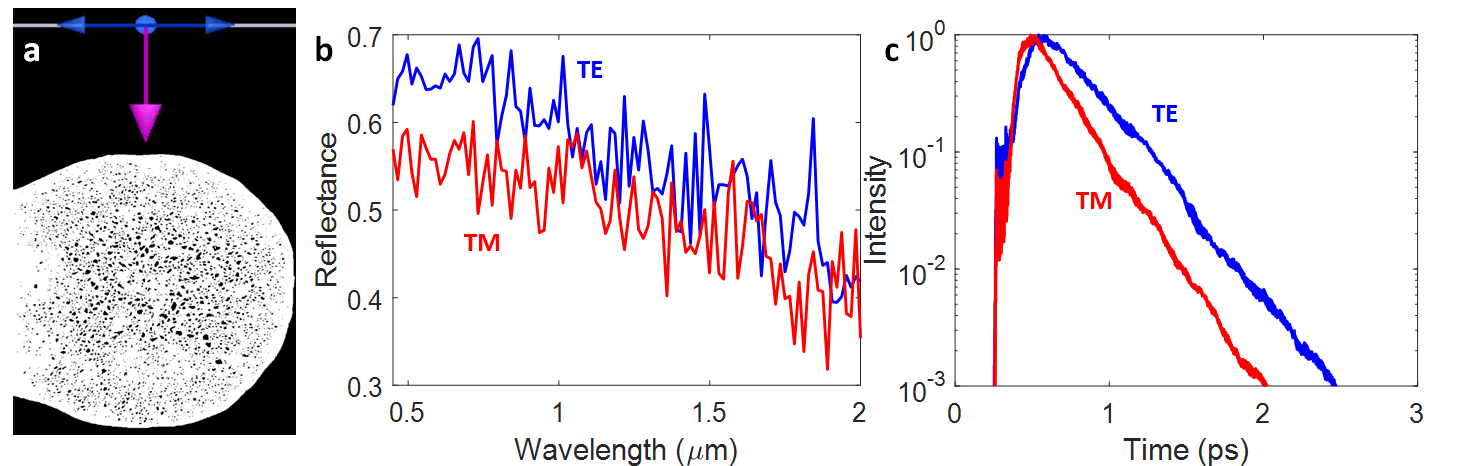
**Figure S2.** (a) Structure extracted from a cocoon fiber and used in FDTD simulations. (b) Simulated reflectance spectra of a single cocoon fiber with TE and TM polarized excitations. (c) Simulated temporal profiles of an ultrashort pulse with TE and TM polarizations passing through a single cocoon fiber.

**5. Time-of-flight measurements**

The photon lifetime of ultra-short pulses passing through the cocoon fibers was characterized using the time-of-flight measurement technique. Two ultra-short pulsed laser beams, the probe beam and the reference or gate beam, were used. A schematic of the experimental setup is illustrated in Fig. S3. A 50× long-working-distance objective was used to focus the probe beam (λ = 600 nm) onto a single cocoon fiber. An imaging arm was added to ensure proper alignment between the input beam and the fiber. The light that passes through the fiber was collected using a parabolic reflector. The collected signal and the reference beam (λ = 800 nm) were focused and spatially and temporally superpositioned onto a Beta Barium Borate (BBO) crystal. The generated sum-frequency signal (λ = 342.86 nm) passed through a narrow bandpass filter and was collected with a photomultiplier tube (PMT). By varying the delay between the two beams, the temporal profile of the probe beam after its interaction with the fiber can be reconstructed. Instrument response function (IRF) of the experimental setup was similarly obtained, where the probe beam did not interact with any specimen.

The probe beam and reference beam have very similar gaussian shaped temporal profiles, and therefore the cross-correlation of the two pulses should also be represented by a gaussian function. A gaussian function was first used to fit the temporal profile of the cross-correlation between the reference and the probe beam before interacting with the fiber specimen. The fitted function was then convolved with an exponential decaying function with a time constant τ to fit measured temporal profiles of TE and TM polarized pulses after exiting the cocoon fiber. The best fit yields photon lifetime τ, which characterizes the strength of light scattering inside the random structures of the cocoon fiber.
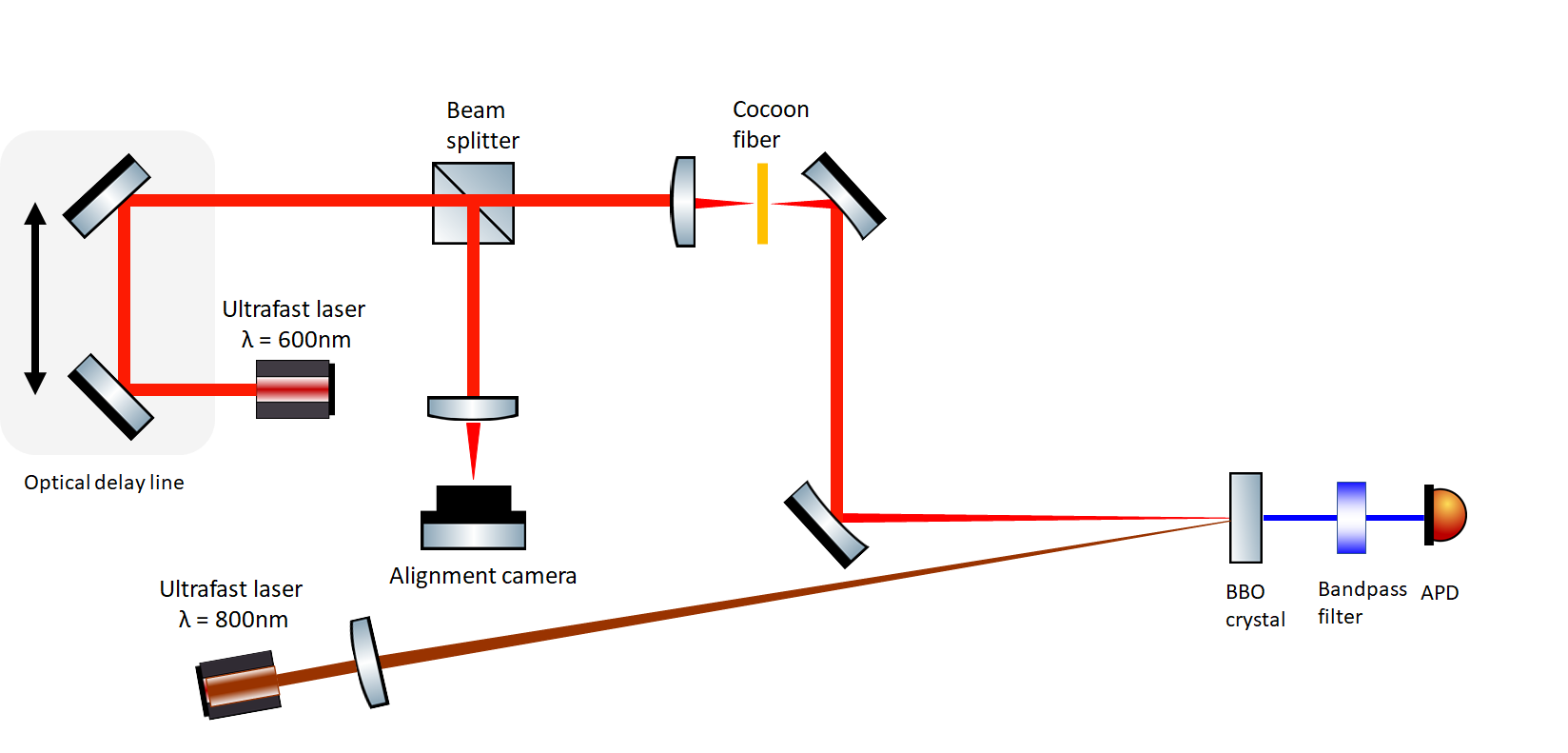


**Figure S3.** Schematic diagram of the time-of-flight measurement technique.

**6. Far-field scattering pattern characterization of single cocoon fibers**

The far-field scattering pattern of cocoon fibers was obtained with a HeNe laser at λ = 633 nm. The beam from the laser was loosely focused onto a single cocoon fiber using a plano-convex lens (f = 25 mm). The far-field scattering pattern of the transmitted light was then captured using a CCD camera. A variable neutral density filter was used to maintain the peak intensity at below 80% of the saturation level of the camera for all the images collected.


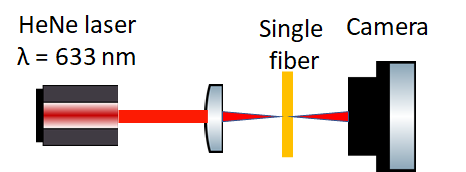


**Figure S4.** Schematic diagram of the far-field scattering pattern characterization technique.

**7. Fabrication of biomimetic fibers using silk fibroin and PVDF**

Degummed *Bombyx mori* silk fibers were obtained and used as the starting point of the fibroin preparation process.^[3]^ A solution of 9.3-M lithium bromide (LiBr) was used to dissolve the silk fibers. A 20% weight-to-volume ratio (i.e., 1 g VS 4 ml) between silk fibroin and the LiBr solution was used to prepare a fibroin-LiBr solution. The fibroin-LiBr solution was left to completely dissolve in an oven set to 60˚C for 4 hours. The dissolved solution was transferred to a dialysis cassette (10 ml, 3500 MWCO), and dialyzed against deionized water for 48 hours. A centrifuge step was used to remove impurities that were left in the solution. The fibroin-LiBr solution was further concentrated with a second set of dialysis cassettes (3 ml, 10000 MWCO), where the solution was dialyzed against a 10% Polyethylene glycol (PEG, 20 kDa) solution for 14-20 hours to achieve the desired concentrations (12-18%).

PVDF solutions were prepared by dissolving Poly (vinylidene fluoride-co-hexafluoropropylene) (PVDF-HFP) in Dimethylacetamide (DMA), with a weight ratio of 1:5 between PVDF-HFP and DMA.

Both silk fibroin and PVDF biomimetic fibers were fabricated using wet spinning. In the case of regenerated silk fibers, 30% ammonium sulfate solution was used as the coagulant, while in the case of PVDF fibers, deionized water was used as the coagulant. A syringe pump connected to a 28-gauge stainless steel, blunt-tip needle was used to extrude the solutions. As the solution entered the coagulation bath, phase separation and polymer precipitation occurred, where a polymer lean phase and a polymer rich phase were generated in the fiber. Eventually the portion of the fiber with polymer lean phase became the voids. These newly solidified porous fibers were spun onto a motorized drum at a controlled speed to achieve the desired fiber diameters. The aspect ratio of the voids could be modified through a draw-down process using a second motorized drum at a higher speed compared to the first drum, where a certain degree of elongation of the voids could be achieved inside the fiber.

Silk solutions at various concentrations (12.7, 13.9, 14.5, and 18.9%) were extruded into the coagulation bath at 20 ml/hr. The speeds of the take-up drum and draw-down drum were set at 4-8 m/min and 10-20 m/min, respectively. PVDF solutions were extruded into the coagulation bath at 10 ml/hr. The speeds of the take-up drum and draw-down drum were set at 1-2 m/min and 2-4 m/min, respectively.


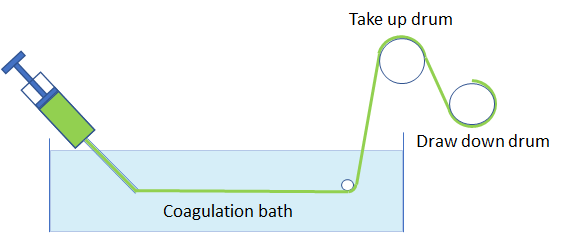


**Figure S5.** Schematic diagram of the apparatus used for wet spinning of biomimetic fibers.

**8. Characterization of fibers supporting transverse Anderson localization**

A supercontinuum laser (SuperK extreme EXU-6) coupled to a filter-based monochromator (λ= 400 -1050 nm, LLTF Contrast) was used as the light source. Light was focused onto the entrance facet of one cocoon fiber with a 50× long-working-distance objective (Mitutoyo 50× M Plan APO). An imaging arm branched out with a removable 50/50 beam splitter was used to align the incident beam with the entrance facet of the fiber. A matching objective, coupled to an imaging camera, was used to image the intensity distribution on the exit facet of the fiber. A variable neutral density filter was used to maintain the peak intensity at below 80% of the saturation level of the camera for all the images collected. The cocoon fibers and the exit facet imaging arm of the setup were mounted on separate XYZ linear translation stages to allow independent alignment and focusing adjustments with respect to the incoming beam.


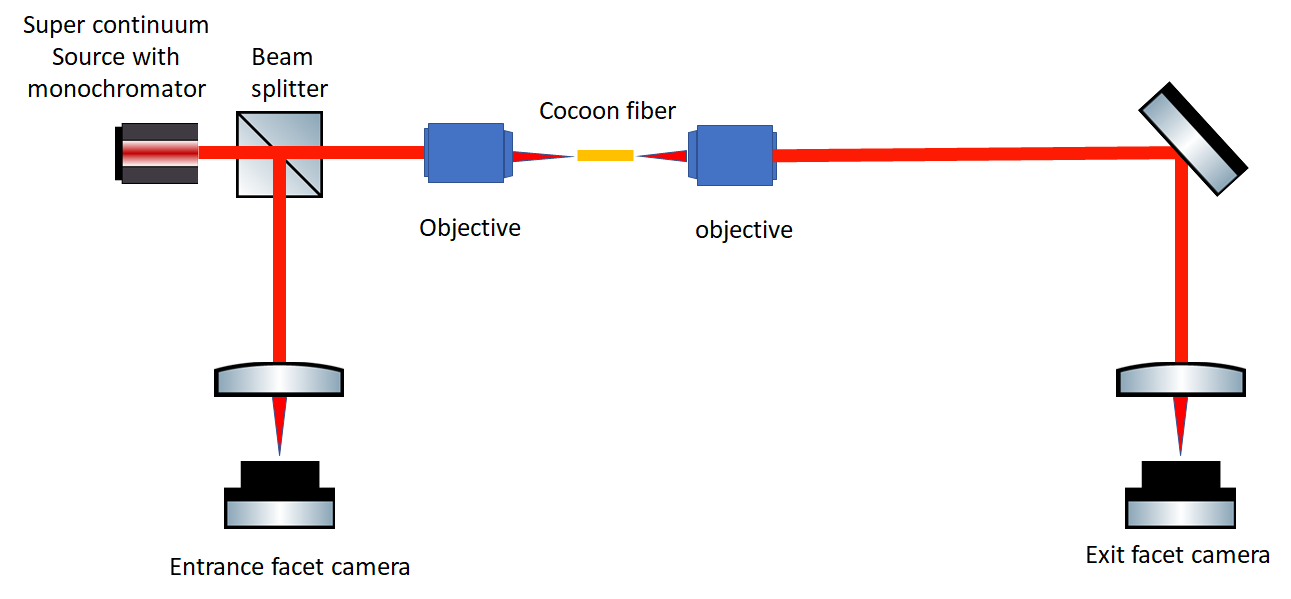


**Figure S6.** Schematic diagram of the optical setup used to characterize light transport in cocoon fibers supporting transverse Anderson localization.

**9. Solar reflectance study on biomimetic fibres with high and low void concentrations**

To further clarify the correlation between void concentrations and fibre reflectance over the solar spectrum, we fabricated regenerated silk fibres and PVDF fibres with both high and low void concentrations and measured their reflectance over the solar spectrum (Fig. S7a). The reflectance measurements clearly show that PVDF and regenerated silk fibres with high void concentrations are significantly more reflective than fibres with low void concentrations. The correlation between void concentration and reflectance is further confirmed by the dark field optical microscopy images of fibres with high and low void concentrations (Fig. S7b), which show that fibres with high void concentrations have a bright, diffused white shine, a clear indication of strong optical scattering. PVDF and regenerated silk fibres with low void concentrations, on the other hand, appear to be semi-transparent as a result of low concentration of scattering centers.

The void concentration of the regenerated silk fibres was controlled by the initial concentration of the regenerated silk solution, as described in section 7 of the supplementary information. Concentrations of 13.9% and 18.9% were used to fabricate fibres with high and low void concentrations, respectively (Figs. S7e,f). The void concentration of the PVDF fibres was controlled by the amount of time the fibres stay in the coagulation bath after they have been drawn. The fibres with high void concentrations used here did not go through a coagulation bath, while the fibres with low void concentrations were soaked in the coagulation bath for 40 hours immediately after the fibre was drawn (Figs. S7c,d).


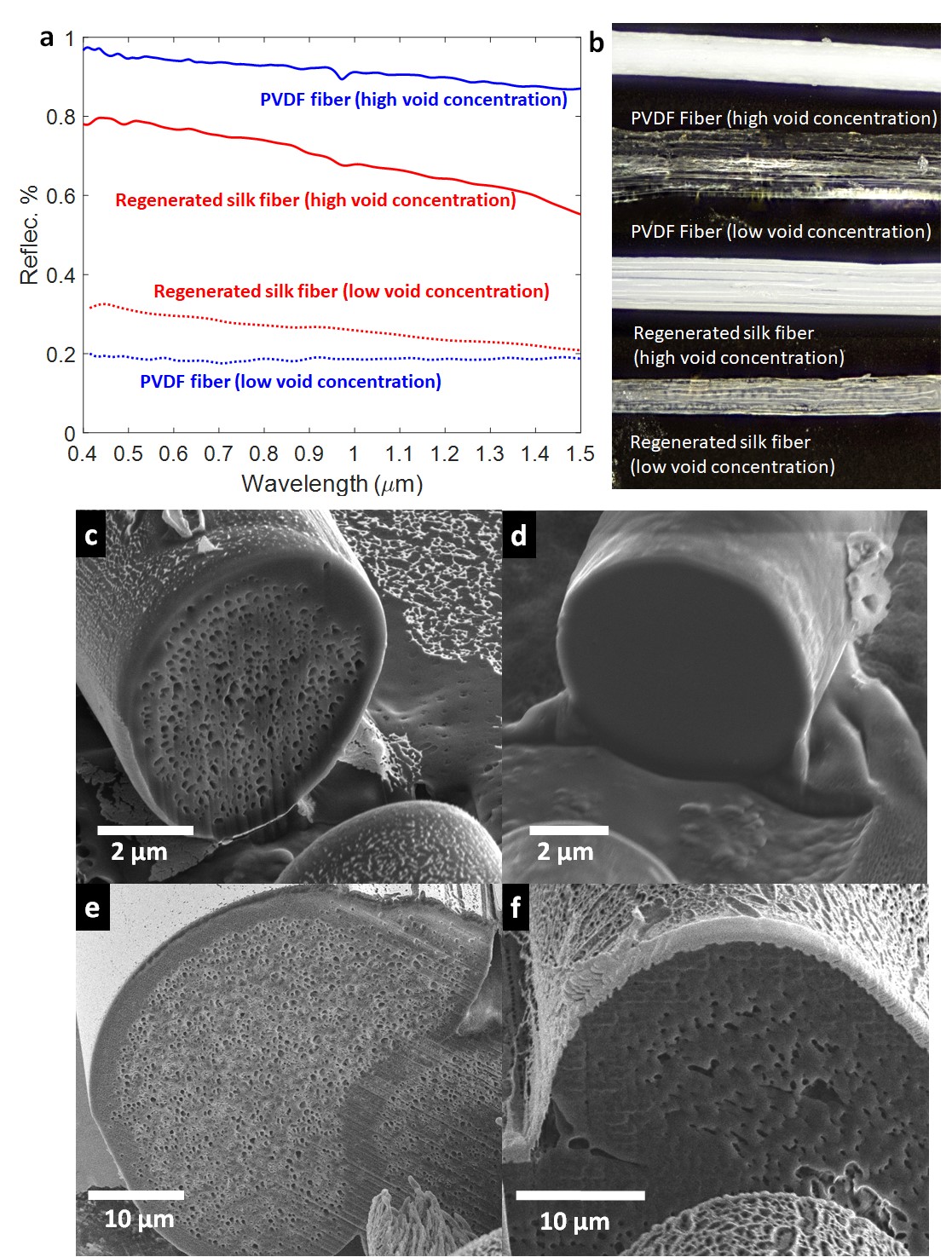
**Figure S7**. Comparative study of biomimetic fibres with high and low void concentrations. (a) Integrated hemispherical reflectance in the visible and near-infrared (λ = 0.4 – 1.5 μm) for regenerated silk and PVDF fibres with high and low void concentrations. (b) Dark field optical microscopy images of regenerated silk and PVDF fibres with high and low void concentrations. (c) and (d) Cross-sectional SEM images of PVDF fibres with high and low void concentrations, respectively. (e) and (f) Cross-sectional SEM images of regenerated silk fibroin fibres with high and low void concentrations, respectively.

**Supplementary References**

[1] Gueymard, C. A., Myers, D. & Emery, K. Proposed reference irradiance spectra for solar energy systems testing. *Sol. Energy* **73**, 443-467 (2002).

[2] Perotto, G. *et al*. The optical properties of regenerated silk fibroin films obtained from different sources. *Appl. Phys. Lett.* **111**, 103702 (2017).

[3] Rockwood, D. N. *et al*. Materials fabrication from Bombyx mori silk fibroin. *Nat. Protoc.* **6**, 1612-1631 (2011).
